# *Mapuera virus*, a rubulavirus that inhibits interferon signalling in a wide variety of mammalian cells without degrading STATs

**DOI:** 10.1099/vir.0.82579-0

**Published:** 2007-03

**Authors:** K. Hagmaier, N. Stock, B. Precious, K. Childs, L.-F. Wang, S. Goodbourn, R. E. Randall

**Affiliations:** 1 School of Biology, University of St Andrews, Fife KY16 9TS, UK; 2 Division of Basic Medical Sciences, St George's, University of London, London SW17 0RE, UK; 3 CSIRO Livestock Industries, Australian Animal Health Laboratory, Geelong, VIC 3220, Australia

## Abstract

*Mapuera virus* (MPRV) is a paramyxovirus that was originally isolated from bats, but its host range remains unknown. It was classified as a member of the genus *Rubulavirus* on the basis of structural and genetic features. Like other rubulaviruses it encodes a V protein (MPRV/V) that functions as an interferon (IFN) antagonist. Here we show that MPRV/V differs from the IFN antagonists of other rubulaviruses in that it does not induce the proteasomal degradation of STAT proteins, key factors in the IFN signalling cascade. Rather, MPRV/V prevents the nuclear translocation of STATs in response to IFN stimulation and inhibits the formation of the transcription factor complex ISGF3. We also show that MPRV/V blocks IFN signalling in cells from diverse mammalian species and discuss the IFN response as a barrier to cross-species infections.

## INTRODUCTION

*Mapuera virus* (MPRV) was isolated in 1979 from an asymptomatic fruit bat, *Sturnira lilium*, in the Brazilian rainforest. Morphological studies indicated that it was a paramyxovirus (Zeller *et al.*, 1989). Subsequent analysis of its protein expression pattern and the sequence of the nucleoprotein (NP) gene further classified MPRV as a member of the genus *Rubulavirus* (Zeller *et al.*, 1989). Although MPRV is not thought to be pathogenic for humans, intracranial infections of mice were shown to be fatal (Zeller *et al.*, 1989). Its natural host range, however, is unknown.

The V/P gene of rubulaviruses encodes two proteins, V and P, but no C proteins. V is produced by translation of a faithful mRNA copy of the V/P gene. Expression of the phosphoprotein P requires the introduction of G nucleotides into the transcript, a process directed by stuttering of the virus polymerase at an editing motif in the virus genomic RNA (Thomas *et al.*, 1988). Consequently, the two translation products share a common N-terminal domain, while their C-terminal domains, downstream of the editing site, are unique. Paramyxovirus V proteins show significant sequence similarity, especially in the C-terminal V-unique region which contains seven highly conserved cysteine residues that bind two zinc atoms (Liston & Briedis, 1994; Paterson *et al.*, 1995).

Following virus infection *in vivo*, the interferon (IFN) response is critical in delaying virus spread and buying time for the adaptive immune system to control the infection. Cells of all tissues react to viral infections by activating signalling cascades that lead to the induction of type I IFN. The newly synthesized IFN is secreted and binds to the IFN-*α*/*β* receptor on the surface of the infected cell and of neighbouring cells, inducing a Jak/STAT signalling cascade. STAT1 and STAT2 (signal transducers and activators of transcription) are activated by phosphorylation and form heterodimers that associate with a third factor, p48 [also called IFN regulatory factor 9 (IRF-9) or ISGF3*γ*]. The resulting complex, IFN-stimulated gene factor 3 (ISGF3), translocates to the nucleus and, through a promoter element called ISRE (IFN-stimulated response element), induces the transcription of IFN-stimulated genes (ISG), many of which have antiviral activity. Certain immune cells secrete gamma IFN (IFN-*γ*) in response to virus infections. IFN-*γ* signalling also involves a Jak/STAT signalling cascade. When activated by phosphorylation, STAT1 forms a homodimeric complex GAF (*γ*-activated factor) that after translocation into the nucleus binds to promoters containing a GAS (*γ*-activated sequence) element, inducing the transcription of various genes that are, among others, involved in the regulation of the adaptive immune response (reviewed by Goodbourn *et al.*, 2000; Haller *et al.*, 2006; Sen, 2001).

Like most other viruses, paramyxoviruses have evolved mechanisms that allow them to at least partially overcome the IFN response in order to establish a productive infection (reviewed by Conzelmann, 2005; Garcia-Sastre, 2004; Horvath, 2004; Nagai & Kato, 2004; Stock *et al.*, 2005). They achieve this by both reducing the production of IFN by infected cells and blocking IFN signalling. The V proteins of many paramyxoviruses, including parainfluenza virus 5 (PIV5, previously referred to as *simian virus 5* or SV5; Chatziandreou *et al.*, 2004) and MPRV, are able to reduce the induction of IFN in response to dsRNA or virus infection (Andrejeva *et al.*, 2004; Childs *et al.*, 2006; He *et al.*, 2002; Poole *et al.*, 2002; Wansley & Parks, 2002). Central to this is the interaction of V proteins with mda-5 (melanoma differentiation-associated gene-5) (Andrejeva *et al.*, 2004; Childs *et al.*, 2006). mda-5, like its homologue RIG-I (retinoic acid-inducible gene-I), is a DEAD box helicase that functions as a sensor for dsRNA and promotes activation of the IRF-3 and NF-*κ*B signalling cascades that ultimately induce the expression of IFN (reviewed by Haller *et al.*, 2006; Sen & Sarkar, 2005).

In addition, many paramyxoviruses target cellular STAT factors to inhibit the signalling cascade induced by IFN (Horvath, 2004; Nagai & Kato, 2004; Stock *et al.*, 2005). Members of the genera *Morbillivirus* (Ohno *et al.*, 2004; Palosaari *et al.*, 2003; Shaffer *et al.*, 2003; Takeuchi *et al.*, 2003; Yokota *et al.*, 2003) and *Henipavirus* (Park *et al.*, 2003; Rodriguez *et al.*, 2002, 2003, 2004; Shaw *et al.*, 2004) sequester STAT1 and STAT2 in cytoplasmic or nuclear complexes that affect their localization and, in the case of *Nipah virus* (NiV), also their activation by Tyr-phosphorylation. Most rubulaviruses such as PIV5, *Human parainfluenzavirus 2* (hPIV2) or *Mumps virus* (MuV) have been shown to target one of the STAT factors for proteasome-mediated degradation (Andrejeva *et al.*, 2002a, b; Didcock *et al.*, 1999; Kubota *et al.*, 2005; Parisien *et al.*, 2001; Ulane *et al.*, 2003, 2005; Yokosawa *et al.*, 2002; Young *et al.*, 2000). The molecular mechanism by which the V protein of PIV5 (PIV5/V) induces the degradation of STAT1 has been thoroughly studied. It involves the assembly by V of a complex of cellular factors, including STAT1, STAT2, DDB1 (the 127 kDa subunit of the UV-damaged DNA-binding protein) and Cullin4A (Cul4A, an adaptor protein involved in the formation of ubiquitination complexes) (Andrejeva *et al.*, 2002a; Lin *et al.*, 1998; Parisien *et al.*, 2002b; Precious *et al.*, 2005a, b; Ulane & Horvath, 2002; Ulane *et al.*, 2003). Recently, the structure of this complex has begun to be revealed with publication of the structure for DDB1 and V (Li *et al.*, 2006). The complex constitutes a functional E3 ubiquitin ligase that initiates the polyubiquitination of STAT1. Ubiquitinated STAT1 is thereby marked as a target for the proteasome and degraded. It is likely that additional cellular factors are involved [for example, Ulane *et al*. (2005) have proposed involvement of the RING domain protein Roc1 (regulator of cullins 1)].

More recently, Precious *et al*. (2005a) have characterized the formation of the complex from purified components *in vitro* and propose the following model: PIV5/V is able to interact independently with both STAT2 and DDB1. DDB1 recruits cellular components like Cul4A that are part of the ubiquitination machinery. STAT1 itself does not interact directly with PIV5/V, but as STAT1 and STAT2 form transient heterodimers even under non-induced conditions (Braunstein *et al.*, 2003; Precious *et al.*, 2005a), it is recruited into the complex via its association with STAT2. The loss of STAT1 by degradation destabilizes the interaction of V/DDB1/Cul4A with STAT2, which would facilitate its replacement with a new STAT1/2 heterodimer. The involvement of STAT2 is particularly interesting in this context in that it confers species specificity to the process. Due to the divergence of murine and human STAT2, PIV5 does not induce degradation of STAT1 in murine cells (Young *et al.*, 2001), but is able to use exogenously supplied human STAT2 to form a functional complex that induces the degradation of endogenous murine STAT1 (Parisien *et al.*, 2002a). The inability of PIV5 to block the IFN response in mice is thus one host-range barrier that the virus would need to overcome if it were to adapt to naturally infect mice.

## METHODS

### Cells.

Vero (monkey kidney), HeLa (human cervical carcinoma), Hep2 (human larynx carcinoma), PK15 or PKIBRS2 (porcine kidney), BF (BALB/c fibroblast), NBL6 (horse dermis), MDCK (canine kidney), Tb1-Lu (bat lung fibroblasts) and 2fTGH (human fibrosarcoma) cells were used. Transfections for luciferase reporter assays were carried out using Lipofectamine (Invitrogen); all other transfections were done using FuGENE (Roche).

### Viruses.

PIV5 strain W3A (Choppin, 1964), rPIV5VΔC (He *et al.*, 2002) and MPRV strain BeAnn 370284 (Henderson *et al.*, 1995) were grown and titrated in Vero cells. For virus infections, cell monolayers were incubated for 90 min with an appropriate dilution of virus stock in serum-free Dulbecco's modified Eagle's medium.

### IFN.

Recombinant human IFN-*α*A/D (Rehberg *et al.*, 1982) or recombinant human IFN-*γ* (R&D Systems) was used for stimulation of human and simian cells, and recombinant porcine IFN-*α* or recombinant murine IFN-*α* for porcine or murine cells. For the production of bat, canine or horse IFN, Tb1-Lu, MDCK or NBL6 cells were infected with 10 p.f.u. per cell rPIV5VΔC, washed three times and incubated for 40 h. The supernatant was cleared of debris by centrifugation at 5000 r.p.m. (4500 ***g***) for 15 min, cleared of virus particles by ultracentrifugation at 38 000 r.p.m. (250 000 ***g***) for 6 h, UV-irradiated for 2 min, stored at −70 °C and used for stimulation of the respective cells at a dilution of 1 : 3.

### Plasmids.

The IFN-*α*/*β*-responsive plasmid p(9-27)4tkΔ(−39)lucter contained four tandem repeats of an ISRE sequence fused to the firefly luciferase gene (King & Goodbourn, 1998). The IFN-*γ*-responsive plasmid p(GAS)2tkΔ(−39)lucter contained a minimal thymidine kinase promoter and two tandem repeats of a GAS sequence fused to the luciferase gene (King & Goodbourn, 1998). pJATlacZ contains a *β*-galactosidase gene under the control of the rat *β*-actin promoter (Masson *et al.*, 1992). pEF.plink2, pEF.myc.IRES and pEF.PIV5/V have been described by Didcock *et al.* (1999). A cDNA fragment of MPRV/V (for the sequence see GenBank accession no. EF035449) was amplified by PCR (forward primer: gggccatggacctcaccttctctc; reverse primer: gggtctagatcattcttgatctgattc) and cloned into pEF.myc.IRES for mammalian expression of an N-terminally myc-tagged version under the control of the EF-1*α* promoter. The NiV/V expression plasmid has been described previously (Hagmaier *et al.*, 2006).

### Antibodies.

Anti-STAT1 (sc-417; Santa Cruz Biotechnology), anti-STAT1-Tyr-P (07-307; Upstate), anti-STAT2 [sc-839 (N-terminal) and sc-476 (C-terminal); Santa Cruz Biotechnology], anti-STAT2-Tyr-P (07-224; Upstate), anti-STAT3 (ab2984; Abcam), anti-PIV5/V PK336 (MCA1360; Serotec), anti-MPRV/V (polyclonal mouse serum after immunization with MPRV/V or antibodies from sheep immunized with PIV5/V, affinity purified on MPRV/V), anti-myc (Clone 4A6, 05-724; Upstate, or sc-789; Santa Cruz Biotechnology) and anti-actin (A5441; Sigma-Aldrich) were used.

### Cell lines.

Stable cell lines expressing the V protein of MPRV were produced as described previously (Andrejeva *et al.*, 2002b). Hep2 cells were transfected with pEF.myc.IRES.MPRV, selected in the presence of 400 μg geneticin ml^−1^ and screened for expression of MPRV/V by immunofluorescence.

### IFN signalling assays.

Cells were cotransfected with 350 ng p(9-27)4tkΔ(−39)lucter or p(GAS)2tkΔ(−39)lucter, 350 ng pJATlacZ and 470 ng of a V expression plasmid. Forty-eight hours post-transfection, the cells were either induced or not induced with 1.8×10^4^ IU IFN-*α* ml^−1^ for 4 h. Bat, dog and horse cells were stimulated with IFN supernatant for 6 h. Luciferase and *β*-galactosidase activities were measured as described previously (King & Goodbourn, 1994). Differences in transfection efficiency or cell number were taken into account by dividing the luciferase values by the *β*-galactosidase values. The activity induced in the absence of V protein was set at 100 %.

### Immunofluorescence.

Cells were grown on coverslips, fixed and stained with specific antibodies as described previously (Randall & Dinwoodie, 1986). Antibody binding was visualized using Texas red- or FITC-conjugated immunoglobulins (101002, 401002, 101007 and 401007; Oxford Biotechnology). Cell nuclei were stained using DAPI (Sigma-Aldrich).

### Electrophoretic mobility shift assay (EMSA).

Nuclear extracts were incubated with a radiolabelled ISRE probe and separated on a native polyacrylamide gel as described (Didcock *et al.*, 1999). The dried gels were visualized by autoradiography.

### Recombinant protein capture assay.

MPRV/V was cloned into pGEX4T (Pfizer) modified to include a GST tag and a tobacco etch virus (TEV) protease cleavage sequence N-terminal to the gene of interest (Precious *et al.*, 2005b). GST-PIV5/V has been described previously (Precious *et al.*, 2005b). Recombinant baculoviruses containing human STAT2 and STAT1 genes were the kind gift of Professor I. Julkunen (Department of Microbiology, National Public Health Institute, Helsinki, Finland) (Fagerlund *et al.*, 2002). Baculovirus containing human DDB1 was cloned as described previously (Precious *et al.*, 2005a). *Spodoptera frugiperda* (Sf9) cells were infected with recombinant baculoviruses and capture of proteins expressed in extracts by GST-MPRV/V, GST-PIV5/V or GST alone was carried out essentially as previously described (Precious *et al.*, 2005a), followed by analysis by 4–12 % gradient SDS-PAGE (NuPage; Invitrogen).

## RESULTS

### V protein of MPRV inhibits IFN signalling

**Fig. 1. f1:**
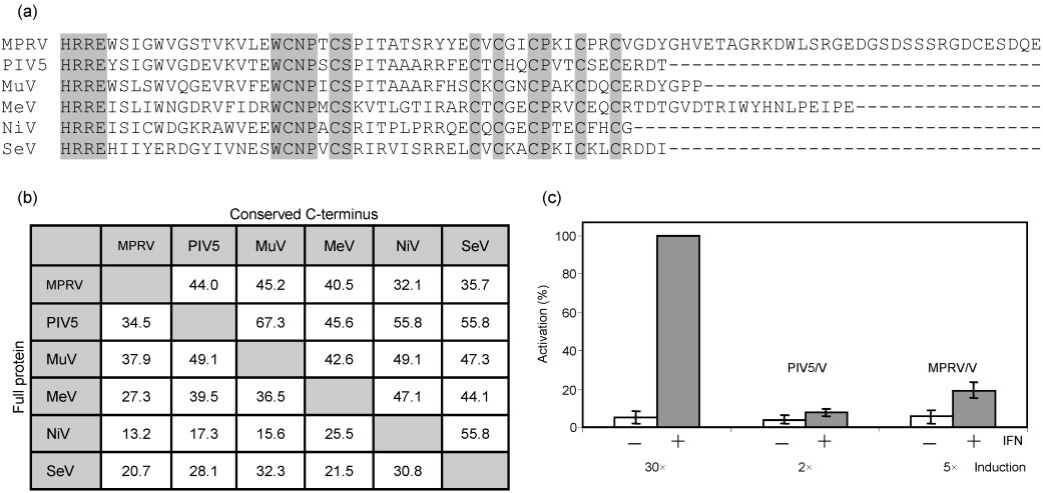
MPRV/V is an IFN antagonist. (a) Alignment (clustalw) of amino acid sequences of the unique C-terminal end of paramyxoviral V proteins. (b) Amino acid similarity (percentage of total residues) of full-length V proteins (left half) or conserved C-terminal ends as shown in (a) (right half). (c) IFN signalling assay. HeLa cells were cotransfected with a luciferase reporter construct under the control of an ISRE promoter, a *β*-galactosidase reporter construct under the control of a constitutive promoter, and an effector plasmid encoding either no protein, PIV5/V or MPRV/V. Forty-eight hours post-transfection, cells were induced or not with IFN-*α* for 4 h. Lysates were analysed for luciferase activity. The data show mean values of three independent experiments. Error bars represent sd. The induction factor (mean of stimulated/unstimulated values) is indicated below each pair of bars.

The V gene of MPRV (MPRV/V) was obtained by RT-PCR from MapV strain BeAnn 370284. The cDNA sequence was determined and deposited in GenBank (accession no. EF035449). As in other rubulaviruses, MPRV/V is translated from the unedited transcript of the genome, while the mRNA for the P protein is generated by non-templated G insertion. Comparison of the MPRV/V protein sequence with that of other paramyxovirus V proteins showed relatively low similarity of the P/V-common domains but high conservation of the V-specific C terminus (see Fig. 1a[Fig f1]), which is to be expected for a paramyxovirus V protein. The diagram in Fig. 1(b) compares three rubulaviruses and three other paramyxoviruses with respect to the similarity of either their full-length V proteins or the conserved C-terminal ends. In accordance with the classification of MPRV as a member of the genus *Rubulavirus*, the sequence of MPRV/V is most closely related to the V sequences of other rubulaviruses. Compared to the V protein of PIV5, MPRV/V contains an additional C-terminal tail of 36 aa, which is the main reason for the fact that the conserved C terminus of PIV5/V has a lower similarity to that of MPRV/V than to that of other rubulaviruses. A database search did not identify any known motifs in this extension. A similar shorter, C-terminal extension is found in the V protein of MeV, but does not show any sequence similarity.

The MPRV/V cDNA was cloned into a mammalian expression vector with an N-terminal myc tag and tested for its ability to block IFN signalling in a reporter assay based on the induction of a luciferase gene in response to stimulation with IFN-*α*/*β*. In human HeLa cells (Fig. 1c[Fig f1]), the ISRE promoter was strongly induced after stimulation with IFN-*α* in the absence of V protein. In the presence of the PIV5/V or MPRV/V protein, the induction of the reporter was almost completely abolished. We also analysed the effect of MPRV/V on IFN-*γ* signalling using a reporter construct that contains a GAS instead of the ISRE promoter. Induction of the reporter in response to IFN-*γ* treatment was inhibited when either PIV5/V or MPRV/V was expressed (not shown), indicating that MPRV is able to block both IFN signalling pathways.

### MPRV/V prevents formation of ISGF3 complex

**Fig. 2. f2:**
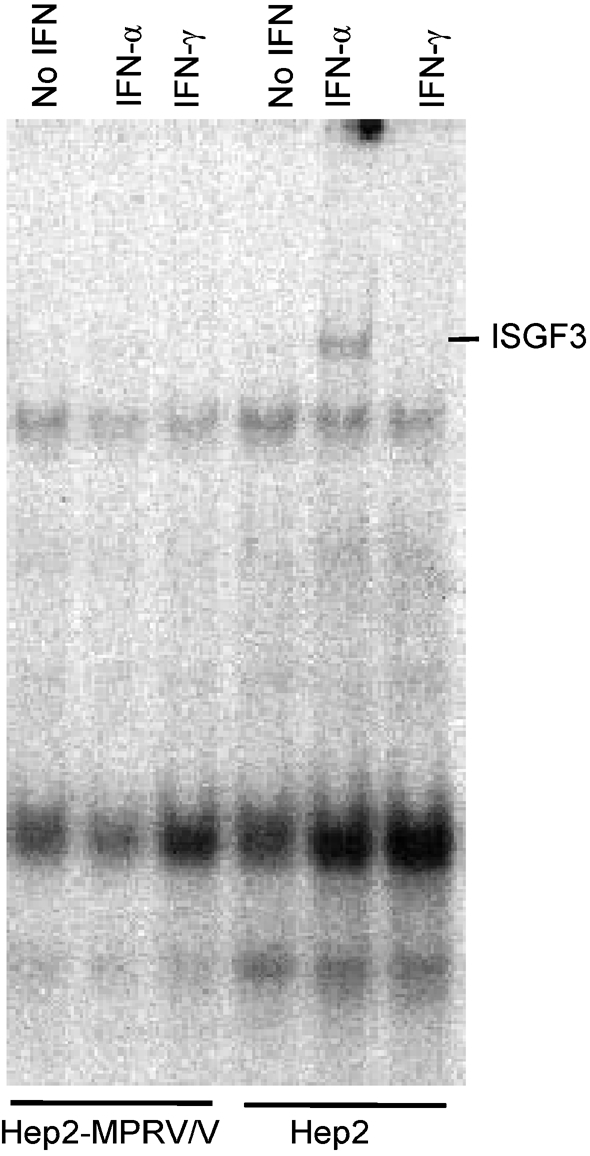
ISGF3 formation in MPRV/V-expressing cells. EMSA of naïve and myc-MPRV/V-expressing Hep2 cells after treatment with 5×10^4^ IU human IFN-*α* or human IFN-*γ* ml^−1^ for 100 min. Nuclear cell extracts were prepared and incubated with a radiolabelled probe from the ISRE of the 9–27 promoter. The ISGF3 complex is indicated to the right of the panel.

The final step in the IFN-*α*/*β* signalling cascade is the binding of ISGF3 to the ISRE promoter element. To investigate whether MPRV/V had an influence on the formation of this complex, a stable Hep2 cell line was generated that expressed MPRV/V. MPRV/V-expressing and naïve cells were stimulated with IFN-*α* or IFN-*γ*. The lysates were incubated with a labelled DNA fragment containing the ISRE sequence and analysed by EMSA for the presence of the ISGF3 complex (Fig. 2). As expected, a shift of the ISRE band could be detected in lysates from naïve IFN-*α*-treated, but not IFN-*γ*-treated or untreated cells. In the MPRV/V-expressing cells, however, IFN-*α* stimulation did not induce a shift of the labelled DNA element, suggesting that MPRV/V interfered with the correct formation of the ISGF3 complex.

### MPRV does not induce STAT degradation

**Fig. 3. f3:**
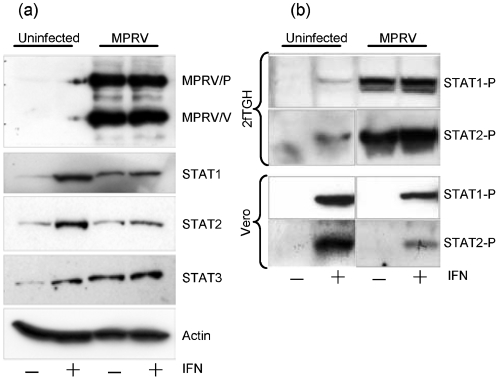
STATs in the presence of MPRV/V. (a) STAT degradation. 2fTGH cells were infected with MPRV strain BeAnn 370284 at 10 m.o.i. or mock-infected. They were stimulated with 3.2×10^4^ U human IFN*α* ml^−1^ at 16 h p.i. or left untreated. At 25 h p.i., the cells were harvested and the lysates were analysed by Western blot probed with monoclonal antibodies against human STAT1, STAT2, STAT3 and cellular actin, as well as with an antiserum raised against the P and V proteins of MPRV. More than 95 % of the cells were infected at the time of harvest, as determined by immunofluorescence. (b) STAT phosphorylation. 2fTGH and Vero cells were infected as above and stimulated for 20 min with 2.5×10^3^ U human IFN-*α* ml^−1^ at 22 h p.i. or left untreated. Cells lysates were analysed by Western blot probed with antibodies against human Tyr701-phosphorylated STAT1 or Tyr689-phosphorylated STAT2.

Although it has been reported that hPIV4 does not block IFN signalling (Nishio *et al.*, 2005), the V proteins of all rubulaviruses examined to date that do block IFN signalling do so by targeting either STAT1 or STAT2 for proteasomal degradation (Andrejeva *et al.*, 2002a, b; Didcock *et al.*, 1999; Kubota *et al.*, 2005; Parisien *et al.*, 2001; Ulane *et al.*, 2003, 2005; Yokosawa *et al.*, 2002; Young *et al.*, 2000). Given that MPRV was a rubulavirus and that it inhibited both IFN-*α*/*β* and IFN-*γ* signalling, we expected its V protein to induce STAT1 degradation. To determine if this was the case, 2fTGH cells were, or were not, infected with MPRV. Immunofluorescence analysis confirmed an infection efficiency of >95 %. At 16 h post-infection (p.i.) the cells were treated or not with IFN-*α* for 9 h. Total cell lysates were analysed by Western blot probed with antibodies specific for the P and V proteins of MPRV as well as STAT1 (Fig. 3a[Fig f3]). Comparable amounts of lysate were loaded in each lane as monitored by detection of cellular *β*-actin (bottom panel). Surprisingly, we found no evidence for the degradation of STAT1 in MPRV-infected cells. Furthermore, when the same lysates were probed with antibodies specific for STAT2 (and STAT3), no reduction in the levels of these proteins was observed. In control cells infected with PIV5, STAT1 was no longer detectable (not shown).

It was also noted in this and similar experiments that, in the absence of exogenous IFN, STAT1 levels were higher in MPRV-infected cells than in unstimulated uninfected cells (STAT1 being upregulated by IFN). These results suggest that some IFN was induced following infection with MPRV and that, at early times p.i., IFN could signal in MPRV-infected cells. However, the virus clearly did inhibit IFN signalling, as the levels of STAT1 in MPRV-infected cells did not increase further upon the addition of exogenous IFN at 16 h p.i. and were significantly lower than in uninfected cells stimulated with IFN.

### MPRV/V does not influence STAT1 phosphorylation

As MPRV was able to block both the IFN-*α*/*β* and IFN-*γ* signalling pathways, though not by depletion of STAT1, we addressed its influence on the IFN-induced phosphorylation of STAT1 at tyrosine residue 701 (Shuai *et al.*, 1993). 2fTGH cells were infected with MPRV, stimulated with IFN-*α* for 20 min and the lysates were analysed by Western blot with antibodies against Tyr701-phosphorylated STAT1 (Fig. 3b[Fig f3]). In untreated cells, no Tyr701-phosphorylated STAT1 could be detected. After IFN stimulation, a doublet band corresponding to phosphorylated STAT1*α* and STAT1*β* was detected both in uninfected and infected cell lysates, indicating that Tyr-phosphorylation of STAT1 was not inhibited by any of the MPRV proteins expressed during infection. Again, some IFN seemed to have been induced following infection as STAT1 was phosphorylated in MPRV-infected cells in the absence of exogenous IFN. Further analysis revealed that MPRV also did not prevent phosphorylation of STAT2 (Fig. 3b[Fig f3]). In addition, it appeared that the phosphorylated forms of both STAT1 and STAT2 were stabilized in MPRV-infected cells, as they were still detectable after 24 h infection. Similar experiments in Vero cells (which are deficient in IFN production) confirmed that MPRV does not inhibit IFN-induced phosphorylation of either STAT1 or STAT2. Phosphorylated STAT1 and STAT2 only became detectable in infected cells after IFN treatment (Fig. 3b[Fig f3], bottom panels).

### MPRV/V interacts with recombinant human STAT1 and STAT2 but not DDB1

**Fig. 4. f4:**
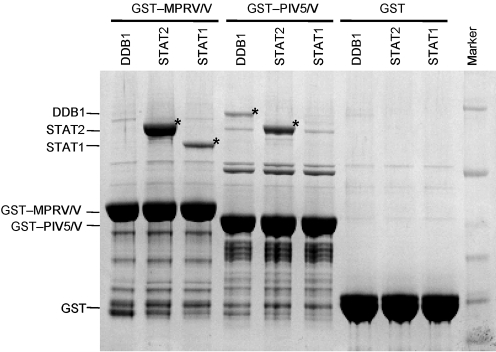
Interaction of MPRV/V with cellular factors. Purified GST-tagged V proteins or GST alone were coupled to glutathione beads and incubated with human DDB1, STAT1 or STAT2 expressed in the baculovirus system. Proteins bound to beads were separated on a 4–12 % gradient SDS-PAGE gel and visualized by Coomassie staining. Bands corresponding to DDB1, STAT1 and STAT2 are marked by asterisks.

Since MPRV did not induce STAT degradation, but clearly blocked IFN signalling, we addressed the question of whether MPRV/V interacted directly with STAT1 and STAT2. Furthermore, given the central role of DDB1 in STAT degradation by the V proteins of PIV5 and PIV2, we also examined the interaction of MPRV/V with DDB1. STAT1, STAT2 and DDB1 were individually expressed from recombinant baculoviruses in Sf9 cells. Soluble cell extracts were mixed with glutathione–agarose beads saturated with bacterially expressed GST, GST–MPRV/V or GST–PIV5/V. Proteins recovered from the glutathione beads were separated by gradient SDS-PAGE and visualized by Coomassie staining (Fig. 4). No specific proteins were captured in the absence of V protein (GST alone). GST–MPRV/V, however, was able to capture both STAT1 and STAT2, but not DDB1. In contrast, and as previously reported (Precious *et al.*, 2005a), GST–PIV5/V is able to bind directly to STAT2 and DDB1, but not STAT1. These observations suggest that the reason why MPRV does not target STAT1 or STAT2 for degradation is its inability to interact with DDB1 to form an E3 ubiquitination complex. These results were supported by a parallel approach based on yeast two-hybrid assays (not shown), in which MPRV/V interacted with STAT1 and STAT2, but not with DDB1, whereas PIV5/V interacted independently with both DDB1 and STAT2 but not STAT1.

### MPRV/V prevents nuclear translocation of STAT1 and STAT2

**Fig. 5. f5:**
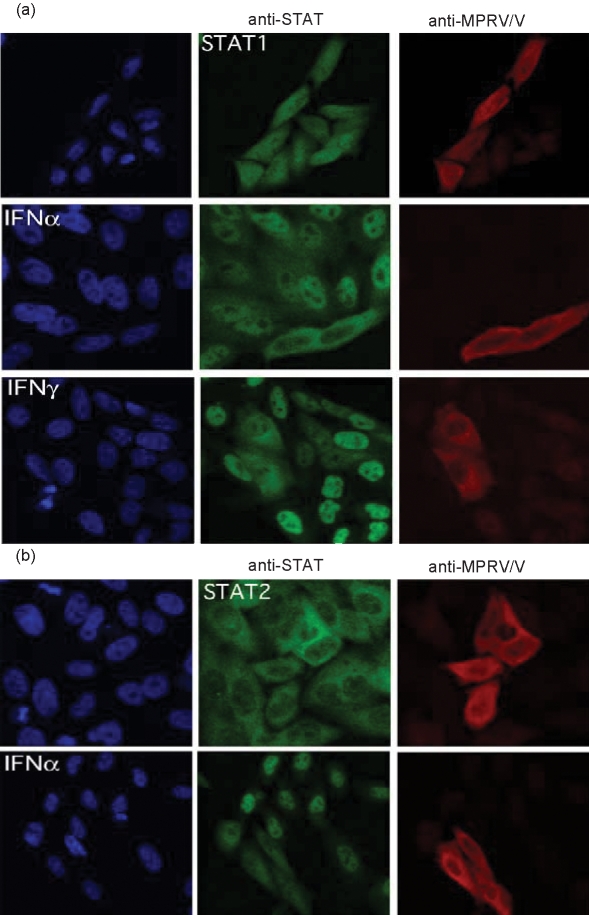
STAT nuclear translocation. Hep2 cells were transfected with a construct encoding myc-tagged MPRV/V. At 48 h p.i., the cells were stimulated with 10^5^ IU human IFN-*α* ml^−1^ for 70 min or left untreated. The cells were analysed by immunofluorescence with antibodies against (a) the V protein of MPRV (red) and STAT1 (green), or against (b) the V protein of MPRV (red) and STAT2 (green).

As MPRV/V was clearly able to bind to both STAT1 and STAT2 and to block their function, but did not affect their stability or activation, the inhibition may simply reflect the physical association of MPRV/V with STATs. The V proteins of henipa- and morbilliviruses have been shown to work in a similar fashion to prevent import of the STATs into the nucleus (Palosaari *et al.*, 2003; Park *et al.*, 2003; Rodriguez *et al.*, 2002, 2003, 2004; Shaw *et al.*, 2004). We therefore examined whether MPRV prevented IFN-induced relocalization of STAT1 and STAT2 into the nucleus. Hep2 cells that had been transfected with a plasmid expressing MPRV/V were stimulated with IFN for 70 min and analysed by immunofluorescence. The cells were stained both with a polyclonal antiserum that recognizes the P and V proteins of MPRV and antibodies against either STAT1 (Fig. 5a[Fig f5]) or STAT2 (Fig. 5b[Fig f5]). While STAT2 was localized in the cytoplasm in the absence of IFN, STAT1, being a shuttling protein in its inactive state (Rodriguez *et al.*, 2004), could be detected both in the nucleus and in the cytoplasm. Treatment with IFN-*α* induced the efficient relocalization of both STATs into the nucleus of untransfected cells. In MPRV/V-expressing cells, however, a clear exclusion of STAT1 and STAT2 from the nucleus was observed following stimulation with IFN-*α*. The nuclear translocation of STAT1 in response to treatment with IFN-*γ* could equally be inhibited by expression of MPRV/V (Fig. 5a[Fig f5], last row). These data strongly suggest that the V protein of MPRV causes incorrect localization of STAT1 and STAT2, thus disrupting both IFN-*α* and IFN-*γ* signalling. The same mislocalization was observed in MPRV-infected cells (not shown).

### MPRV inhibits IFN signalling in a wide range of mammalian cells

**Fig. 6. f6:**
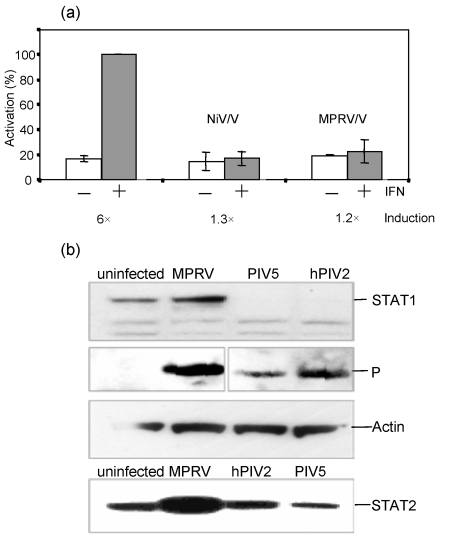
IFN signalling in bat cells. (a) Luciferase reporter assay. Tb1-Lu cells were transfected as described in the legend to Fig. 1(c). NiV/V was used as a positive control. Forty-eight hours p.i., the cells were induced or not for 6 h with bat IFN supernatant, and the lysates analysed for luciferase activity. Data are plotted as described in the legend to Fig. 1(c). (b) STAT degradation. Tb1-Lu cells were infected with MPRV, PIV5 or hPIV2 at 10 m.o.i. or mock-infected. The lysates were analysed by Western blot with antibodies against STAT1, STAT2 and against the P proteins of the different viruses. More than 95 % of the cells were infected at the time of harvest.

Although MPRV/V was able to block IFN signalling, it appeared to do so less efficiently than PIV5, as it allowed for some signalling to occur early in infection. Since we had used mainly human cells throughout this study, but MPRV was originally isolated from fruit bats, we wondered whether the effect of MPRV on the IFN system of bats would be different. We first established whether MPRV/V was able to block IFN signalling in bat cells. The IFN signalling reporter assay described above was adapted to Tb1-Lu lung epithelial cells from *Tadarida brasiliensis*. Because the bat cells were not responsive to commercially available IFN-*α*, they were stimulated instead with supernatant from Tb1-Lu cells that had been infected with rPIV5VΔC, a strong inducer of IFN production. As is evident from Fig. 6(a), MPRV/V inhibited the induction of an ISRE-responsive promoter as efficiently as the V protein of NiV (NiV/V), which was used as positive control.

We next examined whether the virus induced STAT degradation in these cells. Tb1-Lu cells were infected with MPRV, PIV5 or hPIV2 and the levels of STAT1 and STAT2 were monitored by Western blot. Fig. 6(b) (upper panel) shows that while no STAT1 could be detected in cells infected with PIV5, infection with MPRV had not led to degradation of STAT1 in the bat cells. Neither did we notice a reduction in MPRV on the levels of STAT2 in MPRV-infected Tb1-Lu cells (Fig. 6b[Fig f6], bottom panel). Rather, they appeared to be upregulated, as had been noted earlier in IFN-competent human cells. hPIV2 had been included as a control for STAT2 degradation, but induced degradation of STAT1 rather than STAT2 in the bat cells. It has been reported before that hPIV2 can induce degradation of either STAT1 or STAT2 depending on the species of origin (Precious *et al.*, 2005b). It should also be noted that *T. brasiliensis* is an insectivorous bat which is not a host species from which MPRV has been isolated. Unfortunately this is the only bat species from which cultured cells are currently available. Nevertheless, given that we have never observed the degradation of either STAT1 or STAT2 by MPRV in a wide variety of cells, it seems likely that MPRV/V also blocks IFN signalling in cells of its natural host via sequestration of STAT1 and STAT2 in an inactive form in the cytoplasm, rather than targeting them for degradation.

**Fig. 7. f7:**
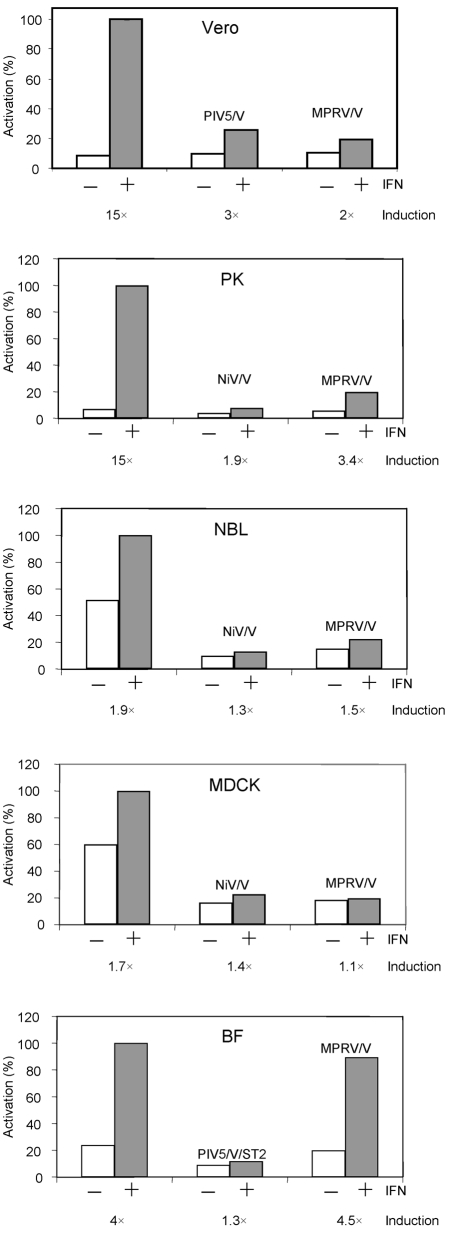
IFN signalling in various species. Representative examples of the IFN signalling assays (performed as for Fig. 1c[Fig f1]) in cells from different mammalian species: Vero (monkey), PK (pig), NBL (horse), MDCK (dog), BF (mouse). Either PIV5/V or NiV/V was used as positive control.

As part of our work on determining the constraints that prevent viruses from crossing species barriers, we were interested in determining whether MPRV/V blocked IFN signalling in cells from different mammalian species. Thus the signalling reporter assay was adapted to cells from a variety of different species and the ability of MPRV/V to block IFN signalling in these cells was analysed. These experiments showed that MPRV/V was able to inhibit IFN-*α*/*β* signalling in human and bat cells (Figs 1c, 6a[Fig f1][Fig f6]), as well as in monkey (Vero), dog (MDCK), horse (NBL-6) and pig (PK15, PKIBRS2) cells, but not in murine (BF) cells (Fig. 7).

## DISCUSSION

The results presented here show that, although MPRV can block IFN signalling, unlike all other rubulaviruses so far examined, it achieves this by sequestering STAT1 and STAT2 in the cytoplasm rather than targeting them for proteasome-mediated degradation. Thus the mechanism of STAT inhibition employed by MPRV shows interesting similarities with morbilli- and henipaviruses, but there are also differences. The V protein of NiV, like MPRV/V, sequesters both STAT1 and STAT2 in the cytoplasm and prevents their import into the nucleus. However, published data suggest that NiV/V is not able to bind both STATs independently. Rather, the interaction of NiV/V with STAT1 is necessary to recruit STAT2 into the complex (Rodriguez *et al.*, 2004). Moreover, the conserved C terminus of NiV/V is dispensable (Park *et al.*, 2003; Shaw *et al.*, 2004), while neither the N-terminal nor the C-terminal domains of MPRV/V are functional on their own (unpublished observations). Also, the V-STAT complex assembled by NiV has been shown to inhibit IFN-induced phosphorylation of STAT1 (Rodriguez *et al.*, 2002), whereas we did not observe an inhibitory effect of MPRV on STAT phosphorylation. On the contrary, the phosphorylated forms of STAT1 and STAT2 that were induced by MPRV infection could still be detected more than 24 h p.i., which would seem to suggest that these forms are stabilized in MPRV-infected cells. This could be due to the fact that MPRV inhibits the import of STATs into the nucleus, where these proteins would normally be dephosphorylated. Like MPRV/V, the V protein of *Measles virus* (MeV/V), the prototype species of the genus *Morbillivirus*, prevents STAT nuclear translocation by simple sequestration in the cytoplasm without apparently affecting its phosphorylation state (Palosaari *et al.*, 2003), although the V protein of the IC-B strain of wild-type MeV has been reported to prevent both STAT1 and STAT2 phosphorylation (Takeuchi *et al.* 2003). However, the N terminus of MeV/V, as well as the P protein of MeV, appears to be functional on their own (Ohno *et al.*, 2004), while expression of the N terminus of MPRV/V was not sufficient to inhibit IFN signalling. The V–STAT complex induced by MPRV does not show strong similarities to its closest relatives, the rubulaviruses, either. PIV5/V does not bind directly to STAT1, but rather uses STAT2 as an adaptor for binding STAT1 (Precious *et al.*, 2005a), while MPRV/V was able to interact with both STAT poteins independently *in vitro*. Despite these differences, analysis of MPRV/V confirmed that it is clearly a rubulavirus V protein. The protein is translated from the unedited mRNA and shows the highest sequence similarity to other rubulavirus V proteins.

There are a number of situations described in which the IFN response acts as a barrier to cross-species infection by paramyxoviruses. For example, one of the reasons why PIV5 is non-pathogenic in mice is because it fails to block IFN signalling in murine cells (Young *et al.*, 2001). Similarly, *Bovine respiratory syncytial virus* may not be pathogenic in humans partially because it fails to successfully overcome the IFN response in human cells (Bossert & Conzelmann, 2002; Young *et al.*, 2003). In the case of MPRV, the results presented here suggest that the intrinsic ability of MPRV to block IFN signalling would not be a significant barrier that prevents this virus from infecting a wide variety of mammalian species. The same also appears to be the case for NiV, as we have recently shown that NiV/V can block IFN signalling in a wide variety of mammalian cells (Hagmaier *et al.*, 2006). However, even if a paramyxovirus such as MPRV is able to block IFN signalling and at the same time to limit IFN production through the interaction of the V protein with mda-5, this does not necessarily mean that the IFN response will not be a barrier to cross-species infection. Thus, we observed a significant upregulation of STAT1 in MPRV-infected human cells (although not as much as in uninfected cells treated with IFN), suggesting that some IFN was induced in response to the infection and that this IFN had activated STAT1 transcription through the Jak/STAT signalling pathway. MPRV-infected cells therefore have time to begin to respond to any small amount of endogenous IFN produced by the infected cells before the virus establishes an efficient block of IFN signalling. It has to be noted in this context that MPRV/V only binds to mda-5, but not to RIG-I, and consequently inhibits only mda-5-mediated, not RIG-I-mediated IFN induction (Childs *et al.*, 2006). As it has been shown that paramyxoviruses can induce IFN expression through a RIG-I-mediated pathway (Kato *et al.*, 2006; Yoneyama *et al.*, 2004), it is reasonable to assume that the IFN response observed in MPRV-infected cells was due to activation of (and failure to inhibit) RIG-I. MPRV clearly did eventually block IFN signalling since the addition of exogenous IFN did not bring the levels of STAT1 up in MPRV-infected cells to those observed in IFN-treated uninfected cells. Thus there is clearly a race between the speed by which a virus can block IFN signalling and the cells' ability to produce and respond to IFN. Whilst there are likely to be many factors which influence the outcome of this race, the balance of these factors may vary from cell to cell and species to species.
